# Synchronous Sinonasal Inverted Papilloma With Nasolacrimal Squamous Cell Carcinoma: An Uncommon Case Report of Malignant Transformation of Inverted Papilloma

**DOI:** 10.7759/cureus.88755

**Published:** 2025-07-25

**Authors:** Julide Kasaboglu, Stoyan Dimitrov, Milena Mitkova, Spiridon Todorov, Tsvetomir Marinov

**Affiliations:** 1 Department of Otolaryngology, Head and Neck Surgery, University Hospital Queen Joanna ISUL, Sofia, BGR; 2 Department of Otolaryngology, Head and Neck Surgery, Medical University-Sofia, Sofia, BGR; 3 Department of Anesthesiology and Reanimation, Medical University-Sofia, Sofia, BGR

**Keywords:** inverted papilloma, lacrimal sac, malignancy, nasolacrimal duct system, sinonasal, squamous cell carcinoma

## Abstract

Nasolacrimal tumors are exceedingly rare head and neck pathologies. They are locally invasive and have an increased possibility of aggressive malignant transformation. These tumors are clinically presented with a palpable mass, obstruction of nasolacrimal drainage, epiphora, and nasal congestion. Nasolacrimal carcinomas are rare malignancies that often take a long time before the correct diagnosis is made. The combination of endoscopic and open en-bloc resection can provide complete removal of locally advanced nasolacrimal tumors. A multidisciplinary team, chemo-radiotherapy, and follow-up monitoring are essential for the effective management of such tumors.

We report a case of a 41-year-old male patient with presentation of epiphora and a paranasal lump. Imaging showed an advanced nasolacrimal tumor with infiltration of surrounding structures. Pathologic examination demonstrated nasolacrimal keratinizing squamous cell carcinoma (SCC) associated with sinonasal inverted papilloma. A multidisciplinary approach, including radical surgery and chemo-radiotherapy, rendered success in achieving remission with a four-year disease-free follow-up.

## Introduction

Sinonasal inverted papilloma (SIP) is a benign tumor that originates from the Schneiderian mucosa in the nasal cavity and accounts for 0.5 to 4% of all sinonasal neoplasms [1,2]. SIP is known for its malignant transformation potential and can massively spread locally. According to the latest literature, the risk of malignant transformation of SIP to squamous cell carcinoma (SCC) is estimated to be around 5% to 15%. Most studies suggest that approximately 10% of SIP cases may undergo malignant transformation into SCC over time. This underscores the importance of surgical excision and ongoing monitoring for patients with SIP to facilitate early detection and management of any malignant changes. The tumors of the nasolacrimal drainage system are exceedingly rare, and since the lacrimal sac and nasolacrimal duct are continuous structures, identification of the primary origin of tumorogenesis becomes more difficult when the staging is enhanced. The risk of malignant degeneration of inverted papilloma into SCC has a very high tendency and both human papillomavirus (HPV) and epidermal growth factor receptor (EGFR) mutations can cause inverted papilloma (IP) transformation to SCC and represent distinct pathways to tumorigenesis. Synchronous and metachronous SCC associated with IP represent distinct patterns of malignant transformation in the sinonasal region. Synchronous SCC refers to the presence of carcinoma and inverted papilloma occurring simultaneously, at the same time, within the same lesion or adjacent sites. In contrast, metachronous SCC develops sequentially, meaning the carcinoma arises after the initial diagnosis and treatment of the inverted papilloma, often months or years later. Synchronous cases may suggest a more aggressive or field-cancerization phenomenon, while metachronous cases underscore the need for long-term follow-up [3]. 

The authors present a case of a 41-year-old male patient with an advanced synchronous nasolacrimal keratinizing squamous cell carcinoma with sinonasal inverted papilloma.

## Case presentation

A 41-year-old male patient was admitted to the Department of ENT for surgical management of a tumor originating from the nasolacrimal system. The patient reported a gradually enlarging lump in the lacrimal sac area, accompanied by persistent epiphora (continuous tearing) on the right side, which he had been experiencing for approximately three years. Over the past year, he also experienced recurrent episodes of epistaxis (nosebleeds) and nasal obstruction, both of which persisted despite the use of nasal decongestants. These symptoms progressively worsened, leading to increased discomfort and impairment of nasal breathing. On clinical examination, the nasal endoscopy revealed a massive tumor mass that obliterated the whole right nasal cavity, including the nasal vestibule. The nasal septum was severely compressed and displaced by the tumor mass contralaterally.

**Figure 1:**
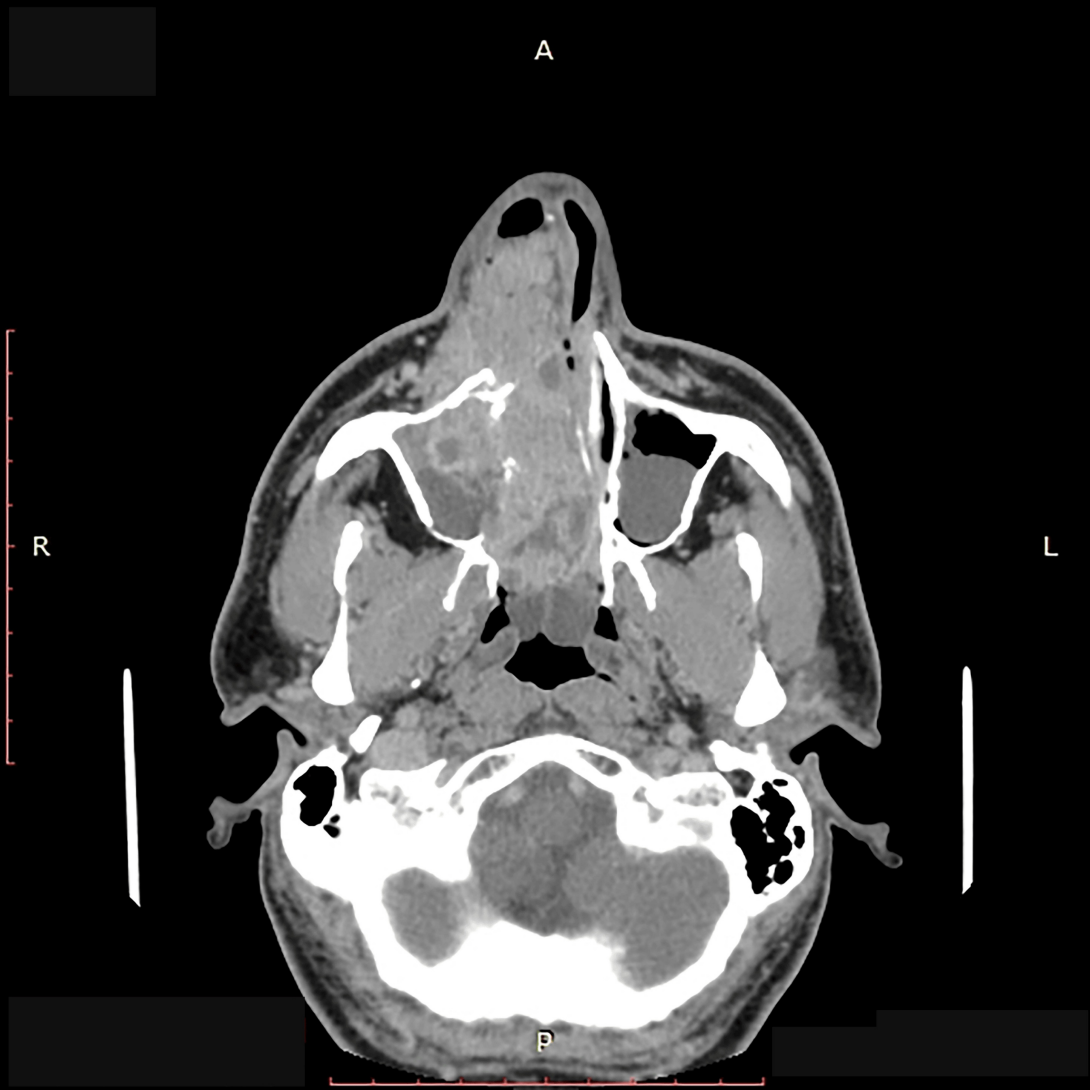
Computer tomography (CT) scan of the paranasal cavities L-left; R-right Tumor infiltration with bone destruction of the medial wall of the maxillary sinus, the frontal process of the maxillary bone, and the medial wall of the orbit, on the right side. The nasal septum was severely compressed and displaced by the tumor mass contralaterally.

**Figure 2:**
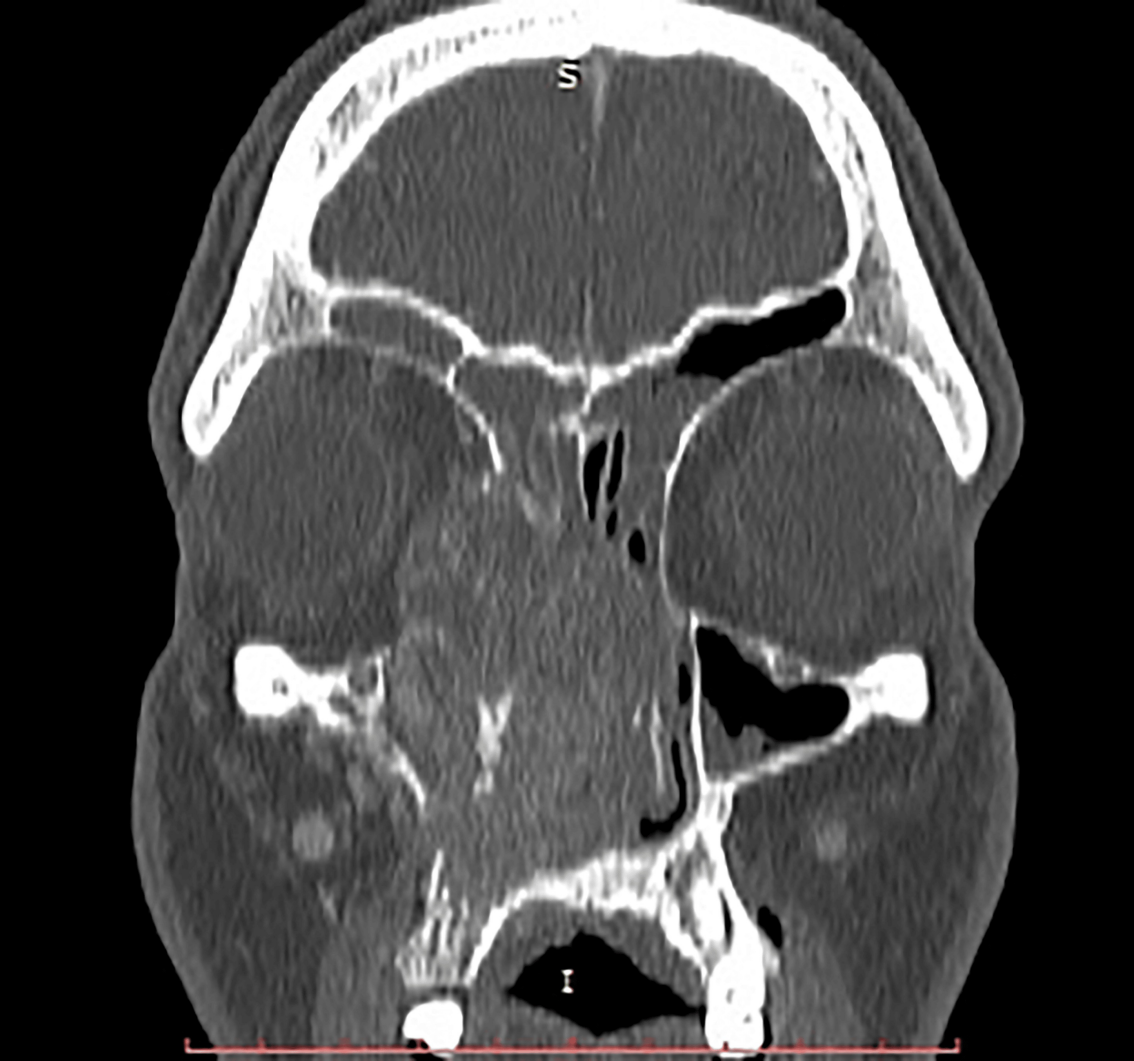
Sagittal view of CT scan of the head The right side of the nasal cavity was filled with tumor mass, which displaced the septum to the left.

A computer tomography (CT) scan of the head showed that a vascularized nasolacrimal tumor mass infiltrated the right lacrimal sac, the lacrimal fossa of the lacrimal bone, and ran downward and slightly laterally, passing through the lacrimal bone and into the right nasal cavity, which was consistent with the facial lump. Significant tumor infiltration was present in the right orbit, the maxillary sinus, and the soft tissues of the buccal region. There were CT signs of bone destruction of the medial wall of the maxillary sinus, the frontal process of the maxillary bone, and the medial wall of the orbit. The right side of the nasal cavity was filled with tumor mass, which displaced the septum to the left (Figures 1, 2). No CT signs of regional or distant metastases were found (T4a N0 M0, Stage III). 

According to the American Society of Anesthesiologists (ASA) Physical Status Classification System, preoperative status was recorded as ASA III, which indicates a severe accompanying disease process.

**Figure 3:**
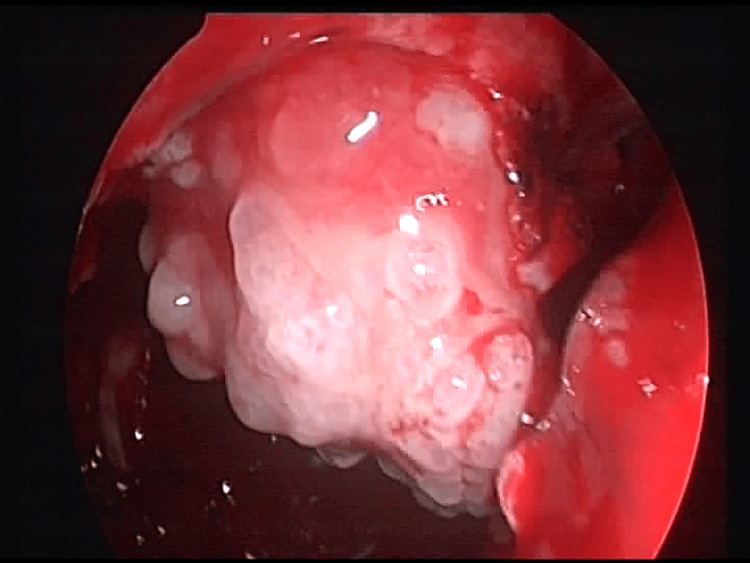
Part of the endoscopic resection of the advanced nasolacrimal tumor with infiltration of surrounding structures.

Medial maxillectomy was performed along with anterior ethmoidectomy. Additional resection of the anterior part of the lamina papyracea, the inferomedial orbital strut, the frontal process of the maxilla, and the medial part of the orbital floor was necessary because of tumor invasion (Figure 3). The lacrimal sac was severely dilated and filled with a grainy tumor mass. Two tissue samples from the maxillary sinus and the lacrimal sac were sent for frozen section biopsy - both showed squamous cell carcinoma. From that point, we proceeded with mediofacial resection since the endoscopic approach alone could not achieve complete surgical removal. The lacrimal sac and nasolacrimal duct, the soft tissues of the cheek, and the orbit, engaged with the tumor, were resected. 

During the early and late postoperative period, no significant complications were observed. Final pathohistology showed low keratinization squamous cell carcinoma, associated with inverted papilloma with clear margins.

Post-operatively, the oncological board appointed the subsequent treatment plan, which included combined chemo-radiotherapy. During the four-year follow-up period till the current date, no signs of disease recurrence were observed. Endoscopic examination, MRI, and Positron-Emission Tomography-CT (PET-CT) scans were used for routine check-ups. Written informed consent was obtained from the patient, who agreed to take part in the study.

## Discussion

IP is an exophytic growth of thickened squamous epithelium and can proliferate into the sinonasal cavity and adjacent structures, more often arising from the maxillary sinus and ethmoidal recess. IP is common in the fifth to eighth decades of human life [1]. The incidence of malignancy transformation is 2% for the inverted type of Schneiderian papilloma [2]. Macroscopically, IP has the appearance of a firm unilateral mass in the nasal cavity with a multi-nodular, erratic surface. Microscopically, the epithelium of the lesion varies in cellularity and typically is composed of squamous, transitional, and columnar cells (sometimes all three types may be present together) along with goblet cells and intraepithelial cystic formations [4,5]. It is clinically presented with complaints such as nasal obstruction, mass formation, unilateral polyp patterns, epistaxis, nasal discharge, and pain. The etiology of inverted papilloma is reported to be linked with viral infections, chronic inflammation, pollution, and occupational exposures such as organic solvents and welding fumes [6]. It is associated strongly with HPV infection which is typically verified by using in situ hybridization or polymerized chain reaction techniques [7,8]. There are also discrepant reports on the presence of Epstein-Barr virus (EBV) in sinonasal inverted papillomas [9,10].

The majority of malignancies are squamous cell carcinomas with keratinization. Seldom, the differential diagnosis may include verrucous carcinoma, mucoepidermoid carcinoma, small cell carcinoma, adenocarcinoma, and undifferentiated carcinoma [3,11]. Malignancy is synchronous with papillomatous lesions. Malignancies may extend to the orbit, lacrimal sac, intracranial cavity, and skull base. Furthermore, invasion of the orbit is highly expected for inverted papilloma. Elner et al. reported a series of 10 patients who were diagnosed with malignant transformation of inverted papilloma. Six of them were with SCC and four of them were diagnosed with transitional cell carcinoma [12]. Nasal congestion, epiphora, proptosis, eye pain, eyelid swelling, and headache are the main presenting symptoms of sinonasal tumor masses invading the orbit [13]. Johnson et al. reported that sinonasal carcinomas with orbital invasion are caused by tumor extension through lamina papyracea in most cases arising from the ethmoidal recess [14]. The most prominent and rare characteristic of the presented case is the location of origin which is the lacrimal sac. Malignant lacrimal sac tumors are rarely documented but could have an aggressive clinical course and poor prognosis. Lacrimal sac SCCs are often initially misdiagnosed as chronic dacryocystitis, whereas early diagnosis is pivotal for the effectiveness of the treatment [13,14]. Song et al. reported a classification with four stages of lacrimal sac malignancies depending on the invading extension of the tumor. Stage I indicates a tumor that is restricted to the lacrimal sac fossa with no palpable mass or no invasion of the surrounding tissues. Stage II includes the infiltration to the naso-lacrimal duct, lacrimal canaliculi, lacrimal caruncula or palpebral conjunctiva. Stage III includes invasion of the nasal cavity/nasolacrimal canal/ethmoid sinus/sphenoid sinus/maxillary sinus/frontal sinus, peripheral bone, or the skin. Finally, stage IV would include infiltration to the orbital apex, meninges/brain, lymph node, or distant metastasis [15].

Depending on the stage, the treatment process consists mainly of surgical intervention and adjuvant therapy for lacrimal sac malignancies. Preferred surgical treatment is complete excision of the lesion with adjacent uninvolved mucosa. Viable surgical approaches comprise open lateral rhinotomy and medial maxillectomy with en bloc excision. The surgical approach should be chosen following the area of origin and attachment and is aimed at reducing the recurrence rate of the sinonasal tumor lesion as well. Mitigating the risk of recurrence in cases of nasolacrimal squamous cell carcinoma involves wide excision of the tumor with clear margins, including the removal of the entire affected lacrimal sac and adjacent involved tissues. Ensuring complete removal of the tumor tissue reduces residual malignant cells that could lead to recurrence. Surgeons often perform meticulous debridement of the tumor margins, sometimes utilizing intraoperative frozen section analysis to confirm the absence of residual disease at the margins. In cases where the carcinoma involves or is close to critical structures, en bloc resection with a safe margin is preferred. Additionally, combining surgery with postoperative radiotherapy can further reduce the likelihood of local recurrence, especially in cases with aggressive features or close margins. Endoscopic resection is a very common and modern technique with safe and excellent results. Furthermore, endoscopic resection is the preferred approach with successful results for lateral and ventrolateral lesions in the nasolacrimal tract [16,17]. Postoperative treatment includes radiation therapy and, in advanced cases, chemotherapy according to the staging and the pathological subtype.

## Conclusions

This is a case of a rare locally advanced synchronous nasolacrimal keratinizing squamous cell carcinoma, associated with inverted papilloma, that took four years from the initial symptoms to the correct diagnosis. With the combination of endonasal and external en bloc resection, we aimed to effectively achieve complete surgical removal with clear margins. These tumors tend to exhibit aggressive behavior, requiring comprehensive management strategies that include surgical excision, radiotherapy, and potentially targeted therapies, as in our case. Accurate diagnosis through histopathological and radiological evaluation is crucial for optimal treatment planning. A multidisciplinary approach remains key to improving outcomes for patients with this unusual malignancy.

## References

[1] Pitak-Arnnop P, Bertolini J, Dhanuthai K, Hendricks J, Hemprich A, Pausch NC (2012). Intracranial extension of Schneiderian inverted papilloma: a case report and literature review. Ger Med Sci.

[2] Wood JW, Casiano RR (2012). Inverted papillomas and benign nonneoplastic lesions of the nasal cavity. Am J Rhinol Allergy.

[3] Eide JG, Welch KC, Adappa ND, Palmer JN, Tong CC (2022). Sinonasal inverted papilloma and squamous cell carcinoma: contemporary management and patient outcomes. Cancers (Basel).

[4] Anari S, Carrie S (2010). Sinonasal inverted papilloma: narrative review. J Laryngol Otol.

[5] Lisan Q, Laccourreye O, Bonfils P (2016). Sinonasal inverted papilloma: from diagnosis to treatment. Eur Ann Otorhinolaryngol Head Neck Dis.

[6] Sun Q, An L, Zheng J, Zhu D (2017). Advances in recurrence and malignant transformation of sinonasal inverted papillomas. Oncol Lett.

[7] Lawson W, Schlecht NF, Brandwein-Gensler M (2008). The role of the human papillomavirus in the pathogenesis of Schneiderian inverted papillomas: an analytic overview of the evidence. Head Neck Pathol.

[8] Loeb KR, Asgari MM, Hawes SE (2012). Analysis of Tp53 codon 72 polymorphisms, Tp53 mutations, and HPV infection in cutaneous squamous cell carcinomas. PLoS One.

[9] Fernandes Q, Merhi M, Raza A (2018). Role of Epstein-Barr virus in the pathogenesis of head and neck cancers and its potential as an immunotherapeutic target. Front Oncol.

[10] Prabhu SR, Wilson DF (2016). Evidence of Epstein-Barr virus association with head and neck cancers: a review. J Can Dent Assoc.

[11] Wenig BM (2009). Undifferentiated malignant neoplasms of the sinonasal tract. Arch Pathol Lab Med.

[12] Elner VM, Burnstine MA, Goodman ML, Dortzbach RK (1995). Inverted papillomas that invade the orbit. Arch Ophthalmol.

[13] Chaudhry IA, Taiba K, Al-Sadhan Y, Riley FC (2005). Inverted papilloma invading the orbit through the nasolacrimal duct: a case report. Orbit.

[14] Johnson LN, Krohel GB, Yeing M (1984). Sinus tumors invading the orbit. Ophthalmology.

[15] Song X, Wang J, Wang S, Wang W, Wang S, Zhu W (2018). Clinical analysis of 90 cases of malignant lacrimal sac tumor. Graefes Arch Clin Exp Ophthalmol.

[16] Villaret AB, Lombardi D, Schreiber A, Farina D, Nicolai P (2013). Oncocytic carcinoma of the nasolacrimal duct treated by transnasal endoscopic resection. Head Neck.

[17] Tomenzoli D, Castelnuovo P, Pagella F (2004). Different endoscopic surgical strategies in the management of inverted papilloma of the sinonasal tract: experience with 47 patients. Laryngoscope.

